# Mercurial Tremor Treated by Strychnine

**Published:** 1855-11

**Authors:** 


Mercurial Tremor treated by Strychnine.—M. Trousseau, the eminent physician of the Hotel Dieu, was lately induced to use strychnine in a case of mercurial tremor, by the good effects of that alkaloid in chorea. Sulphur baths, simple warm baths, opiates, &c., &c., had been used without success; the strychnine was then given in the following manner:—On the first day the patient took a spoonful of a syrup, containing one-fifth of a grain of sulphate of quinine; on the second day two spoonsful, one in the morning and the other at night. No physiological effects ensued. On the third and fourth day the patient took three spoonfuls per diem, at equal intervals in the course of the twenty-four hours. From that time he began to feel some stiffness in his limbs, and this increased on the fifth day, when four spoonfuls were taken. At last the dose was carried to *one grain*, which the man took for two days. On the expiration of the second of these two days, the patient made an effort to rise from his bed; but he had hardly lost hold of the railings when he was seized by a tetanic fit, and hurled like a projectile to the distance of a few steps, his face coming in contact with the ground. On the following days the doses were gradually diminished, and the mercurial tremor (which had become much less immediately the first physiological effects of the strychnine became apparent) completely disappeared on the tenth day after the commencement of the treatment by strychnine. The patient rested a few days without taking any medicinal substances, and left the hospital in excellent condition.—*Ibid.*

